# Left atrial appendage occlusion for recurrent stroke while on oral anticoagulation: a case series

**DOI:** 10.1093/ehjcr/ytae157

**Published:** 2024-03-25

**Authors:** Gonçalo Costa, Mafalda Griné, Mariana Simões, Manuel Oliveira-Santos, Luís Paiva, Marco Costa, Lino Gonçalves

**Affiliations:** Serviço de Cardiologia, Centro Hospitalar e Universitário de Coimbra, Praceta Professor Mota Pinto, 3004-561 Coimbra, Portugal; Faculdade de Medicina da Universidade de Coimbra, Azinhaga de Santa Comba, Celas, 3000-548 Coimbra, Portugal; Serviço de Cardiologia, Centro Hospitalar e Universitário de Coimbra, Praceta Professor Mota Pinto, 3004-561 Coimbra, Portugal; Serviço de Cardiologia, Centro Hospitalar e Universitário de Coimbra, Praceta Professor Mota Pinto, 3004-561 Coimbra, Portugal; Serviço de Cardiologia, Centro Hospitalar e Universitário de Coimbra, Praceta Professor Mota Pinto, 3004-561 Coimbra, Portugal; Faculdade de Medicina da Universidade de Coimbra, Azinhaga de Santa Comba, Celas, 3000-548 Coimbra, Portugal; Serviço de Cardiologia, Centro Hospitalar e Universitário de Coimbra, Praceta Professor Mota Pinto, 3004-561 Coimbra, Portugal; Serviço de Cardiologia, Centro Hospitalar e Universitário de Coimbra, Praceta Professor Mota Pinto, 3004-561 Coimbra, Portugal; Serviço de Cardiologia, Centro Hospitalar e Universitário de Coimbra, Praceta Professor Mota Pinto, 3004-561 Coimbra, Portugal; Faculdade de Medicina da Universidade de Coimbra, Azinhaga de Santa Comba, Celas, 3000-548 Coimbra, Portugal; Coimbra Institute for Clinical and Biomedical Research (iCBR), Coimbra, Portugal

**Keywords:** Atrial fibrillation, Embolic stroke, Anticoagulants, Left atrial appendage closure, Case report

## Abstract

**Background:**

Clinical practice guidelines recommend oral anticoagulation (OAC) for stroke prevention in selected patients with atrial fibrillation (AF). However, some patients still experience thrombo-embolic events despite adequate anticoagulation. The optimal management of these cases remains uncertain, leading to practice pattern variability. We present a series of three cases illustrating the use of left atrial appendage occlusion (LAAO) as an adjunctive stroke prevention strategy in AF patients with recurrent thrombo-embolic events despite adequate anticoagulation.

**Case summary:**

Case one describes an 89-year-old female on apixaban who presented with a thrombus and underwent successful mechanical thrombectomy. Left atrial appendage occlusion was performed, and no subsequent thrombo-embolic events were reported. Case 2 involves a 72-year-old female on full-dose apixaban who experienced recurrent strokes despite adequate anticoagulation. Thrombectomy was performed twice, and complications arose during LAAO. The patient was discharged on warfarin + clopidogrel and remained event-free at the six-month follow-up. Case 3 features an 88-year-old female on rivaroxaban who experienced recurrent cerebral ischaemic events and gastrointestinal bleeding. Left atrial appendage occlusion using an Amplatzer Amulet™ device was successful, and the patient remained event-free at the one-year follow-up.

**Discussion:**

This case series emphasizes the complexity of stroke prevention in AF patients and underscores the need for an individualized approach. Incorporating LAAO alongside OAC can provide additional stroke protection for patients with inadequate response to anticoagulation. Further randomized controlled trials are needed to evaluate the efficacy and safety of this approach. In light of the limited evidence available, these cases contribute to the growing body of knowledge on the potential role of LAAO in secondary stroke prevention in AF patients with recurrent thrombo-embolic events despite appropriate anticoagulation.

**Learning points**

- Left atrial appendage occlusion (LAAO) can be considered as an adjunctive stroke prevention strategy in atrial fibrillation (AF) patients with recurrent thrombo-embolic events despite adequate anticoagulation.
- Individualized management approaches that incorporate LAAO alongside oral anticoagulation can be effective in reducing the risk of recurrent thrombo-embolic events in selected AF patients.

## Introduction

Clinical practice guidelines strongly recommend the use of oral anticoagulation (OAC) for stroke prevention in selected patients with atrial fibrillation (AF).^1,2^ While OAC is highly effective, it does not completely eliminate the risk of stroke, and some patients still experience thrombo-embolic events despite appropriate anticoagulation.^3^ Various strategies have been proposed to address these cases, such as switching to another anticoagulant or incorporating left atrial appendage occlusion (LAAO).^4^ However, due to the limited availability of randomized controlled data supporting these approaches, there is currently no consensus on the optimal management of this clinical challenge, leading to significant variability in practice patterns. In this manuscript, we present a series of three cases that illustrate the use of LAAO as an adjunctive stroke prevention strategy in patients with AF and thrombo-embolic events despite adequate anticoagulation.

## Summary figure

**Table:** 

Time point	Clinical description
**Patient 1**	
November 2022	Acute ischaemic stroke on apixaban (appropriate dose reduction)
February 2023	Percutaneous LAAO
June 2023	No follow-up events
**Patient 2**	
June 2019	Acute ischaemic stroke on warfarin (poor INR control)
December 2020	Acute ischaemic stroke on apixaban (single daily dose)
October 2022	Two acute ischaemic strokes, 6 days apart, on apixaban and later dabigatran (both full dose)
December 2022	Percutaneous LAAO and systemic embolism complication
June 2023	No follow-up events
**Patient 3**	
January 2021	Acute ischaemic stroke on rivaroxaban 15 mg i.d.
April 2021	Acute ischaemic stroke on apixaban 5 mg b.i.d.
April 2022	Gastrointestinal bleeding secondary with diverticular disease
May 2022	Percutaneous LAAO
June 2023	No follow-up events

INR, international normalized ratio; LAAO, left atrial appendage occlusion.

## Patient 1

**Figure 1:**
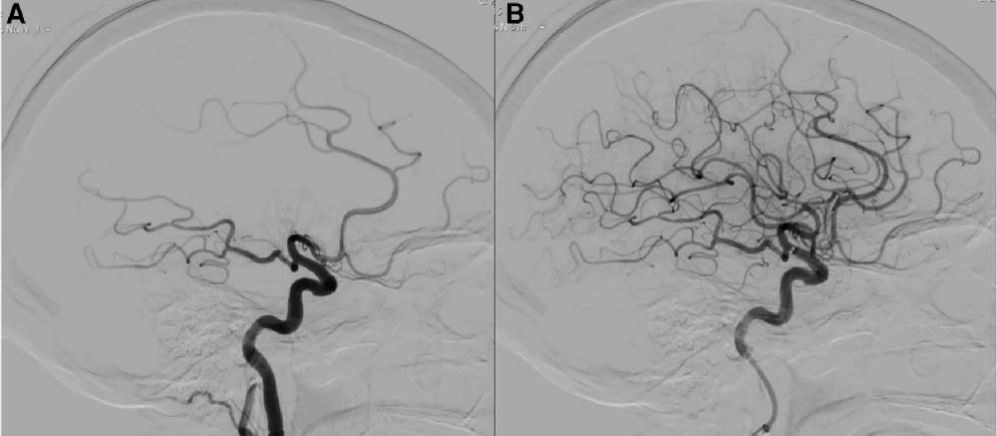
Cerebral angiography. (*A*) Before primary thrombectomy. (*B*) After successful primary thrombectomy with remarkable recovery.

**Figure 2:**
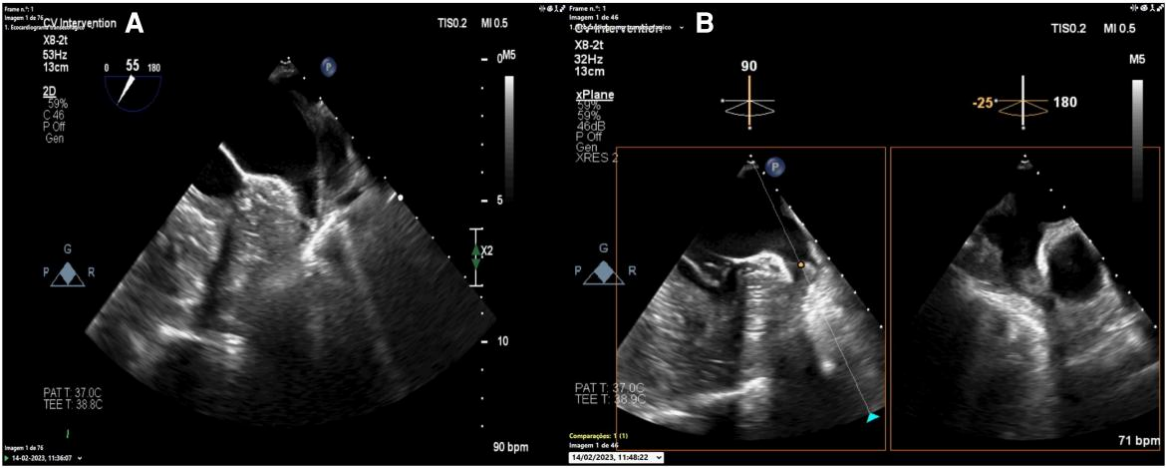
Intraprocedural transoesophageal echocardiography. (*A*) No thrombus in left atrial appendage before femoral puncture. (*B*) Thrombus in formation in the left atrial appendage was detected after femoral puncture demonstrating the high prothrombotic risk of this patient.

**Figure 3:**
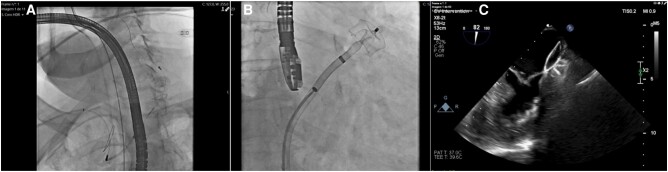
Percutaneous left atrial appendage occlusion. (*A*) SENTINEL™ (Boston Scientific, MA, USA) deployment. (*B*) Amplatzer Amulet™ (Abbott, IL, USA) deployment. (*C*) Transoesophageal echocardiography revealing good final result.

An 89-year-old female with a medical history of hypertension, chronic kidney disease, and non-valvular AF on apixaban (2.5 mg b.i.d., due to creatinine clearance < 30 mL/min) presented to our emergency department with complaints of left-sided weakness and dysarthria. Her blood pressure was 195/105 mmHg, and her pulse was irregular at 75/min. Physical examination revealed partial hemianopia, right gaze deviation, left hemiparesis, mild-moderate sensory loss, dysarthria, and profound hemi-inattention [National Institutes of Health Stroke Scale (NIHSS) score of 12 upon admission]. A head computed tomography (CT) scan showed no acute haemorrhagic or ischaemic changes [Alberta Stroke Program Early CT Score (ASPECTS) of 10], while CT angiography revealed a thrombus at the right M1–M2 transition (*Figure 1A*). The patient underwent mechanical thrombectomy and was discharged in good general condition with near-complete recovery after 4 days [modified Rankin Scale score of 1 and NIHSS score of 3 at discharge] (*Figure 1B*). Apixaban concentration assay revealed concentration within therapeutic range. The case was discussed among our centre’s Vascular Team, and the patient was referred for percutaneous LAAO in addition to the current oral anticoagulation regimen. Pre-procedural transoesophageal echocardiography (TOE) revealed a multilobulated dilated LAA, with self-contrast. Left atrial appendage occlusion was scheduled after four months later using an Amplatzer Amulet™ device (Abbott, IL, USA). Despite maintaining OAC for the procedure, a thrombus in formation in the left atrial appendage was detected after femoral puncture (*Figure 2*) (Supplementary material online, *Videos S1*, *S3* and *S5*). A cerebral protection with SENTINEL™ (Boston Scientific, MA, USA) was used during the occlusion (*Figure 3A*). There were no complications (*Figure 3B* and *C*), and the patient was discharged the following day. As of the present date (approximately four months after LAAO), there have been no documented instances of new thrombo-embolic events.

## Patient 2

**Figure 4:**
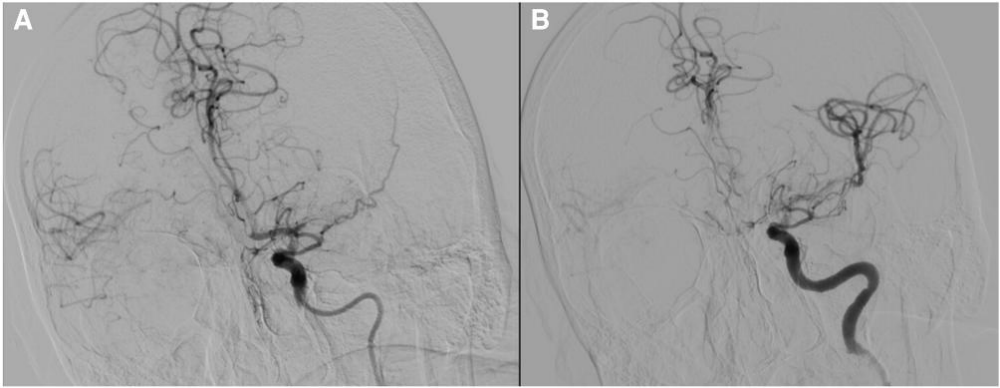
Cerebral angiography. (*A*) Proximal left M2 segment occlusion. (*B*) Result after mechanical thrombectomy.

**Figure 5:**
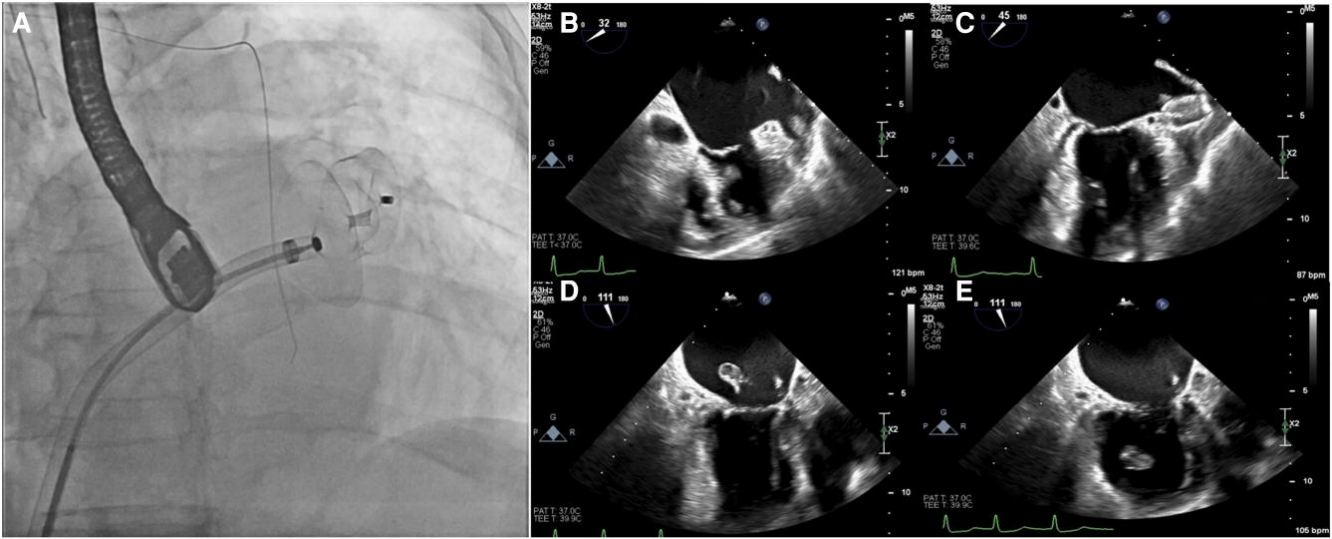
Intraprocedural transoesophageal echocardiography. (*A*) Left appendage occlusion device deployment. (*B*) Thrombus formation in the left atrial appendage before occlude deployment. (*C*) Thrombus attached to occlude device. (*D*) Mobile thrombus in left atria. (*E*) Thrombus advancing to left ventricle with consequent systemic embolism.

**Figure 6:**
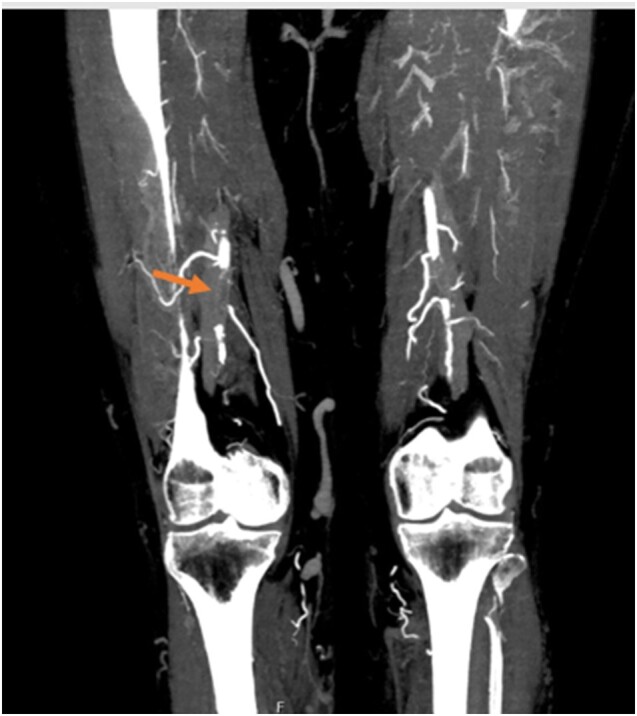
Post-procedural computed tomography angiography revealing a right deep femoral artery thrombus with distal permeabilization.

A 72-year-old female presented to the emergency department with transient aphasia and right-sided weakness. Her medical history included hypertension, permanent non-valvular AF anticoagulated with apixaban, and two previous ischaemic strokes (the first while on warfarin with suboptimal INR, and the second on apixaban 5 mg twice daily). Physical examination did not reveal any significant findings. CT angiography showed a thrombus in the proximal left M2 segment, and the patient underwent successful mechanical thrombectomy (*Figure 4*). Her hospital stay was uneventful, and she was discharged on dabigatran 150 mg twice daily. However, 2 days after discharge, the patient returned with similar complaints, this time without spontaneous resolution. Her blood pressure was 160/77 mmHg, and her pulse was irregular at 102/min. Physical examination revealed right homonymous hemianopia, forced gaze palsy, global aphasia, right hemiplegia, and sensory loss (NIHSS score of 24 upon admission). Non-contrast head CT scan revealed a slight hypodense left lenticular nucleus (ASPECTS of 9) and a hyperdense proximal left M1 segment suggesting acute vessel occlusion (confirmed on CT angiography). Once again, the patient underwent successful primary thrombectomy with remarkable recovery (NIHSS score of 1). Direct OAC measurement was within therapeutic range. Transoesophageal echocardiography during her admission revealed a dilated hypocontractile cauliflower-like LAA and marked spontaneous self-contrast. Patient was discharged on full-dose apixaban. Due to the recurrent events despite adequate oral anticoagulation, the patient was referred for percutaneous LAAO as an adjunctive prevention method. One month later, LAAO with an Amplatzer Amulet™ device was performed (*Figure 5A*). Thrombus formation in the left atrial appendage during the procedure (*Figure 5B* and *C*) (Supplementary material online, *Videos S2* and *S4*) prompted immediate deployment of the SENTINEL™ device. The thrombus then migrated to the left ventricle and subsequently to the systemic circulation (*Figure 5C* and *D*) (Supplementary material online, *Video S6*). Post-procedural CT angiography revealed a deep femoral artery thrombus with distal repermeabilization (*Figure 6*). Since there were no signs or symptoms of acute limb ischaemia, no further interventions were performed, and the patient was discharged on warfarin and clopidogrel (for six months, followed by warfarin alone indefinitely with a target INR of 3–3.5). At the six-month follow-up, no further events were noted.

## Patient 3

**Figure 7:**
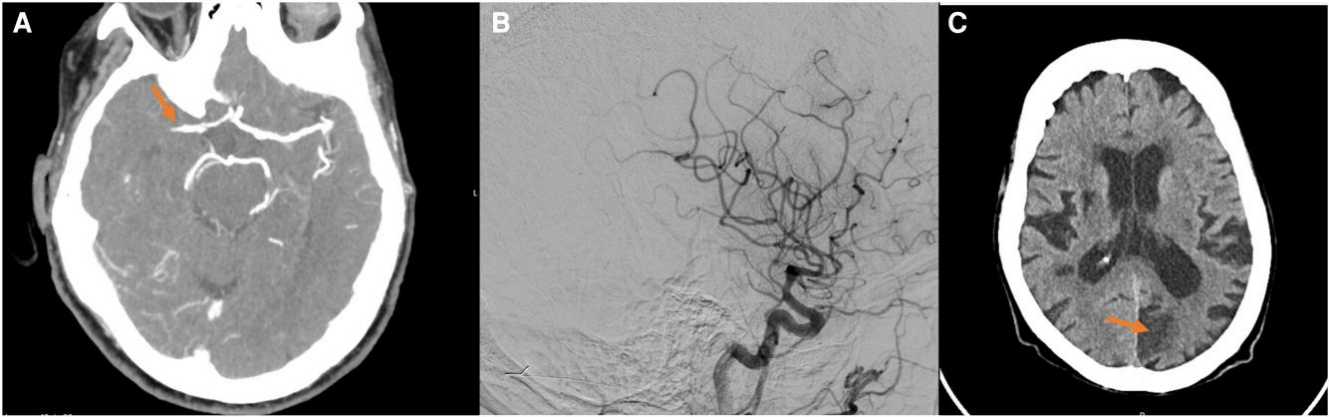
(*A*) Cerebral computed tomography showing presence of a thrombus in the proximal right M1 segment. (*B*) Cerebral angiography after successful thrombectomy. (*C*) Cerebral computed tomography following second stroke 3 months later, exhibiting subacute ischaemic lesion in the left occipital region.

An 88-year-old female patient was admitted with several neurological symptoms, including homonymous hemianopia, left hemi-inattention, and left hemiplegia. Her medical history revealed hypertension, permanent non-valvular AF treated with rivaroxaban 15 mg, and a previous transient ischaemic attack in 2014. CT angiography confirmed the presence of a thrombus in the proximal right M1 segment, leading to the patient undergoing a successful mechanical thrombectomy (*Figure 7A* and *B*). Non-AF stroke aetiologies were excluded. The patient had an uneventful hospital stay and was discharged on apixaban 5 mg twice daily.

**Figure 8:**
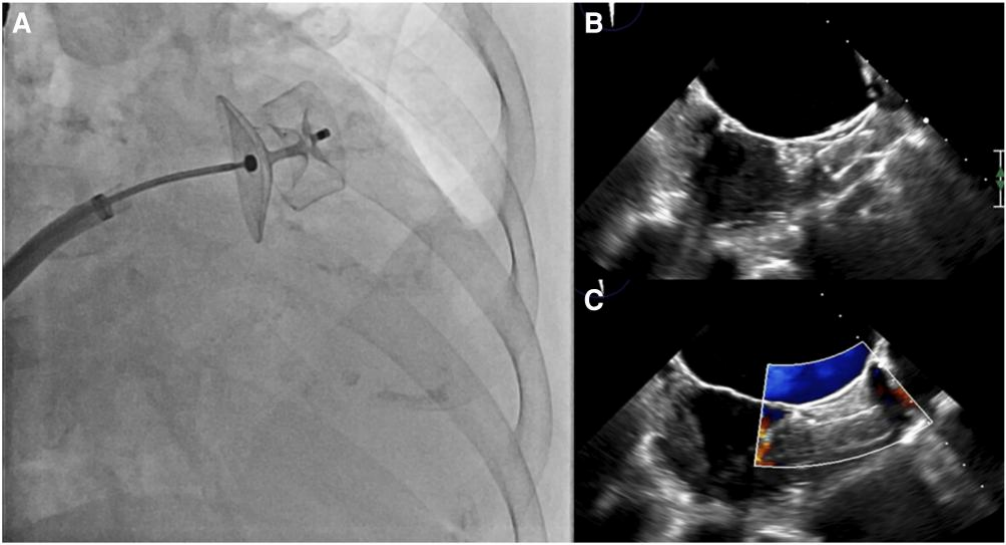
(*A*) Left atrial appendage occlusion device deployment. (*B*) Transoesophageal echocardiography post-procedure with good result without any residual leak.

However, three months later, the patient returned to the emergency department with a severe right hemicranial headache persisting for 2 days. A subacute ischaemic lesion in the left occipital region was identified through cranial CT (*Figure 7C*). Following consultation with the Neurology team, the patient’s oral anticoagulation therapy was switched to edoxaban 30 mg once daily, and she was discharged. Subsequently, due to recurrent cerebral ischaemic events and a new episode of lower gastrointestinal bleeding attributed to diverticular disease, the patient was referred for LAAO. Pre-procedural TOE showed a dilated chicken-wing LAA with self-contrast. The LAAO procedure was successfully performed using an Amplatzer Amulet™ 25 mm device, and no complications were reported (*Figure 8*) (Supplementary material online, *Video S7*). Upon discharge, the patient received double antiplatelet therapy for one month, followed by aspirin 100 mg indefinitely. At the one-year follow-up, no ischaemic or bleeding events were reported.

## Discussion

We present three cases of patients with non-valvular AF experiencing recurrent ischaemic strokes despite adequate OAC and describe the promising role of adjuvant LAAO in this challenging scenario.

Cardioembolic stroke on OAC is an uncommon yet complex event. Firstly, it signals the high-risk nature of this subset of patients, as a previous stroke is one of the strongest predictors of future stroke.^5^ In fact, consistent with previous reports, a recent analysis from COMBINE AF (a dataset of individual patients enrolled in RE-LY, AVERROES, ROCKET AF, ARISTOTLE, and ENGAGE AF-TIMI 48) demonstrated an increased rate of recurrent events and all-cause death, especially in the first year after the index event [pooled cumulative incidence of 7.0% (95% confidence interval 5.2–8.7%) and 18.1% (95% confidence interval 15.7–20.4%), respectively].^3^ Furthermore, proper identification of mechanisms of stroke despite OAC is mandatory to properly manage these patients. The general approach includes identification of non-AF mechanisms, medication error exclusion and imaging exams to exclude intracardiac sources of embolism.^4^ Additionally, routine coagulation tests are of limited utility to assess direct oral anticoagulant (DOAC) efficacy due to their considerable sensitivity variability. Calibrated anti-factor Xa activity, a DOAC-specific coagulation assay, is difficult to interpret as there are no target levels and the absolute values expected at different time points after last DOAC intake are very heterogeneous.^4^

Secondly, the optimal management of such patients is unclear. Traditionally, alternative interventions included intensification of antithrombotic therapy with the addition of an antiplatelet drug or switching to another oral anticoagulant. However, there is little to no evidence to support either of these strategies. Routine addition of an antiplatelet drug has proven deleterious. According to randomized and observational data, there is an increase in bleeding risk with no reduction in stroke.^6,7^ Similarly, though often considered a management option in clinical practice, current observational evidence shows that switching to another OAC is associated with increased risk of stroke recurrence.^8,9^ Network meta-analysis of direct OAC clinical trials rank dabigatran and apixaban as better options for stroke protection, although these findings may not apply to this high-risk population.^10^

The left atrial appendage is the source of most thrombi in patients with non-valvular AF.^11^ The emergence of catheter-based exclusion of the appendage as an alternative to OAC in primary stroke prevention has raised the question of whether LAAO should also be considered in secondary stroke prevention.^12,13^ Indeed, some expert consensuses already contemplate stroke on OAC in their indications for LAAO, provided that adequate OAC use is confirmed and a highly thrombogenic left atrial appendage is present.^14,15^ The latter refers to large, multilobulated, hypocontractile LAAs with significant spontaneous echocardiographic contrast formation or with an actual thrombus despite antithrombotic therapy (referred to as ‘malignant LAA’). This was the case for our patients; hence, they were considered good candidates for the procedure by our centre’s multidisciplinary vascular team. Interestingly, the 2021 LAAOS III trial showed that surgical LAAO in patients with a history of AF undergoing cardiac surgery for another indication provided additional stroke protection when added to OAC (a 33% reduction in the composite endpoint of ischaemic stroke or non-cerebral embolism).^16^ In light of this evidence and contemporary clinical practice, percutaneous LAAO on top of continuous OAC appears to be a viable strategy that merits randomized controlled appraisal.

Lastly, it is important to note that neither OAC nor LAAO addresses other stroke/AF risk factors and comorbid conditions, such as hypertension, obesity, sleep apnoea, or diabetes. Therefore, as highlighted in current guidelines, identification and management of comorbidities are paramount to further reduce the overall risk of stroke, lessen AF burden, and improve cardiovascular outcomes.^17^

In conclusion, this case series demonstrates the intricacy of stroke prevention in some AF patients and highlights the importance of an individualized approach, integrating the possible use of LAAO in the management of patients for whom OAC does not provide sufficient stroke protection.

## Supplementary Material

ytae157_Supplementary_Data

## Data Availability

The data underlying this article will be shared on reasonable request to the corresponding author.

## Lead author biography

Cardiology Resident in Centro Hospitalar Universitário de Coimbra.

## Supplementary material

**Consent:** Consents were obtained in accordance with the Committee on Publication Ethics (COPE) guidelines.

## References

[1] Hindricks G, Potpara T, Dagres N, Arbelo E, Bax JJ, Blomström-Lundqvist C, et al ESC guidelines for the diagnosis and management of atrial fibrillation developed in collaboration with the European Association for Cardio-Thoracic Surgery (EACTS): the task force for the diagnosis and management of atrial fibrillation of the European Society of Cardiology (ESC) developed with the special contribution of the European Heart Rhythm Association (EHRA) of the ESC. Eur Heart J 2020;42:373–498. 10.1093/eurheartj/ehaa612 32860505

[2] January CT, Wann LS, Calkins H, Chen LY, Cigarroa JE, Cleveland JC, et al AHA/ACC/HRS focused update of the 2014 AHA/ACC/HRS guideline for the management of patients with atrial fibrillation: a report of the American College of Cardiology/American Heart Association task force on clinical practice guidelines and the Heart Rhythm Society. J Am Coll Cardiol 2019;74:104–132. 30703431 10.1016/j.jacc.2019.01.011

[3] Benz AP, Hohnloser SH, Eikelboom JW, Carnicelli AP, Giugliano RP, Granger CB, et al Outcomes of patients with atrial fibrillation and ischemic stroke while on oral anticoagulation. Eur Heart J 2023;44:1807–1814. 37038327 10.1093/eurheartj/ehad200 PMC10411934

[4] Galea R, Seiffge D, Räber L. Atrial fibrillation and ischemic stroke despite oral anticoagulation. J Clin Med 2023;12:5784. 37762726 10.3390/jcm12185784 PMC10532406

[5] Lip GY, Nieuwlaat R, Pisters R, Lane DA, Crijns HJ. Refining clinical risk stratification for predicting stroke and thromboembolism in atrial fibrillation using a novel risk factor-based approach: the Euro Heart Survey on atrial fibrillation. Chest 2010;137:263–272. 19762550 10.1378/chest.09-1584

[6] Lip GY . The role of aspirin for stroke prevention in atrial fibrillation. Nat Rev Cardiol 2011;8:602–606. 21788962 10.1038/nrcardio.2011.112

[7] Benz AP, Johansson I, Dewilde WJM, Lopes RD, Mehran R, Sartori S, et al Antiplatelet therapy in patients with atrial fibrillation: a systematic review and meta-analysis of randomized trials. Eur Heart J Cardiovasc Pharmacother 2022;8:648–659. 34142118 10.1093/ehjcvp/pvab044 PMC11905745

[8] Paciaroni M, Caso V, Agnelli G, Mosconi MG, Giustozzi M, Seiffge DJ, et al Recurrent ischemic stroke and bleeding in patients with atrial fibrillation who suffered an acute stroke while on treatment with nonvitamin K antagonist oral anticoagulants: the RENO-EXTEND study. Stroke 2022;53:2620–2627. 35543133 10.1161/STROKEAHA.121.038239

[9] Seiffge DJ, De Marchis GM, Koga M, Paciaroni M, Wilson D, Cappellari M, et al Ischemic stroke despite oral anticoagulant therapy in patients with atrial fibrillation. Ann Neurol 2020;87:677–687. 32052481 10.1002/ana.25700 PMC7383617

[10] López-López J A, Sterne J AC, Thom H HZ, Higgins J PT, Hingorani A D, Okoli G N, et al Oral anticoagulants for prevention of stroke in atrial fibrillation: systematic review, network meta-analysis, and cost effectiveness analysis. BMJ 2017;359:j5058. 29183961 10.1136/bmj.j5058 PMC5704695

[11] Blackshear JL, Odell JA. Appendage obliteration to reduce stroke in cardiac surgical patients with atrial fibrillation. Ann Thorac Surg 1996;61:755–759. 8572814 10.1016/0003-4975(95)00887-X

[12] Holmes DR, Reddy VY, Turi ZG, Doshi SK, Sievert H, Buchbinder M, et al Percutaneous closure of the left atrial appendage versus warfarin therapy for prevention of stroke in patients with atrial fibrillation: a randomised non-inferiority trial. Lancet 2009;374:534–542. 19683639 10.1016/S0140-6736(09)61343-X

[13] Holmes DR J, Kar S, Price MJ, Whisenant B, Sievert H, Doshi SK, et al Prospective randomized evaluation of the Watchman Left Atrial Appendage Closure device in patients with atrial fibrillation versus long-term warfarin therapy: the PREVAIL trial. J Am Coll Cardiol 2014;64:1–12. 24998121 10.1016/j.jacc.2014.04.029

[14] Tzikas A, Holmes DR, Gafoor S, Ruiz CE, Blomström-Lundqvist C, Diener HC, et al Percutaneous left atrial appendage occlusion: the Munich consensus document on definitions, endpoints, and data collection requirements for clinical studies. Europace 2017;19:4–15. 27540038 10.1093/europace/euw141 PMC5841559

[15] Camm AJ . Leap or lag: left atrial appendage closure and guidelines. EP Europace 2023;25:euad067. 10.1093/europace/euad067 PMC10227666 37012659

[16] Whitlock RP, Belley-Cote EP, Paparella D, Healey JS, Brady K, Sharma M, et al Left atrial appendage occlusion during cardiac surgery to prevent stroke. N Engl J Med 2021;384:2081–2091. 33999547 10.1056/NEJMoa2101897

[17] Hindricks G, Potpara T, Dagres N, Arbelo E, Bax JJ, Blomström-Lundqvist C et al 2020 ESC guidelines for the diagnosis and management of atrial fibrillation developed in collaboration with the European Association for Cardio-Thoracic Surgery (EACTS): the task force for the diagnosis and management of atrial fibrillation of the European Society of Cardiology (ESC) developed with the special contribution of the European Heart Rhythm Association (EHRA) of the ESC [published correction appears in Eur Heart J. 2021 Feb 1; 42(5):507] [published correction appears in Eur Heart J. 2021 Feb 1; 42(5):546–547] [published correction appears in Eur Heart J. 2021 Oct 21; 42(40):4194]. Eur Heart J 2021;42:373–498. 32860505 10.1093/eurheartj/ehaa612

